# The impact of vitamin and mineral supplements usage prior to COVID-19 infection on disease severity and hospitalization

**DOI:** 10.17305/bjbms.2021.7009

**Published:** 2022-02-28

**Authors:** Refat M. Nimer, Omar F. Khabour, Samer F. Swedan, Hassan M. Kofahi

**Affiliations:** Department of Medical Laboratory Sciences, Jordan University of Science and Technology, Irbid, Jordan

**Keywords:** COVID-19, supplements, vitamin D, hospitalization, severity

## Abstract

The COVID-19 pandemic has caused a global public health emergency. Nutritional status is suggested to be related to the severity of COVID-19 infection. Herein, we aimed to explore the impact of using vitamin and mineral supplements prior to COVID-19 infection on disease severity and hospitalization. In addition, the prior use of aspirin as an anticoagulant on the disease severity was investigated. A cross-sectional, self-administered survey was conducted between March and July 2021. Recovered COVID-19 individuals (age ≥ 18 years, n = 2148) were recruited in the study. A multivariate logistic regression was used to evaluate the associations of supplements and aspirin use with COVID-19 disease severity and hospitalization status. Among the participants, 12.1% reported symptoms consistent with severe COVID-19, and 10.2% were hospitalized due to COVID-19. After adjustment for confounding variables (age, gender, BMI, cigarette smoking status, and the number of comorbidities), the multivariate logistic regression model showed that the consumption of vitamin D supplements prior to COVID-19 infection was associated with a significant decrease in disease severity (OR = 0.68, 95% CI 0.50-0.92; p = 0.01), and a lower risk of hospitalization (OR = 0.64, 95% CI 0.45-0.89; p = 0.01). On the other hand, there were no significant differences in the frequencies of severe illness and hospitalizations with the consumption of vitamin A, folic acid, vitamin B12, vitamin B complex, vitamin C, zinc, iron, selenium, calcium, magnesium, omega 3, and aspirin before COVID-19 infection. Among the investigated nutrients, the use of vitamin D prior to COVID-19 infection was associated with reduced disease severity and hospitalization. However, more studies are required to confirm this finding.

## INTRODUCTION

The coronavirus disease 2019 (COVID-19) is a worldwide pandemic caused by severe acute respiratory syndrome coronavirus 2 (SARS-CoV-2) [1,2]. According to the WHO, a total of 271,963,258 COVID-19 cases and 5,331,019 deaths were reported as of December 17^th^, 2021 [3]. SARS-CoV-2 infection is associated with a broad spectrum of symptoms ranging from moderate to severe pneumonia, coagulopathy, and death [4]. The percentage of severe cases requiring hospitalization varies from country to country but is estimated to account for 10-20% of all SARS-CoV-2 infections globally [5,6].

Dietary immunomodulators are substances that affect the functions of the immune system. Examples include vitamins A, C, D, E, and beta-carotene, as well as microelements such as zinc, selenium, and omega-3 fatty acids [7,8]. These substances are essential for acquired and innate immunity mediated by neutrophils, macrophages, natural killer cells, and T lymphocytes [7,9,10]. Therefore, deficiency in dietary immunomodulators could increase the risk of severe COVID-19 infection [11]. Such deficiencies can be corrected by following a healthy diet combined with the proper use of supplements. Although the use of vitamin and mineral supplements does not prevent infection with COVID-19 [12], it can improve immune status and subsequent disease severity [13,14]. Hence, the use of supplements has increased drastically during the COVID-19 pandemic [15].

Since COVID-19 has been shown to cause blood clotting, the use of aspirin has increased during the pandemic to overcome such complications. In addition to preventing clotting, aspirin reduces blood levels of interleukin-6 (IL-6), C-reactive protein, and macrophage colony-stimulating factor [16]. Therefore, aspirin use may help in reducing the occurrence of COVID-19-induced thrombosis and cytokine storm [17].

Several studies have reported the benefits of using vitamin and mineral supplements following a diagnosis of COVID-19 to improve the clinical course of the disease [18-21]. However, the benefits of regular intake of supplements before COVID-19 infection on the clinical course remain undefined. The study aimed to investigate the effect of regular use of supplements and aspirin before COVID-19 infection on disease severity and hospitalization status. We hypothesize that using some of these dietary supplements would be associated with reduced disease severity and fewer hospitalizations.

## MATERIALS AND METHODS

### Study design and participants

This study is part of the Jordanian COVID-19 survey project (JCSP). Ethical approval for the project was obtained from the institutional review board of Jordan University of Science and Technology (Ref.: 3/139/2021, dated: 30/03/2021). This cross-sectional study was conducted in Jordan between March and July 2021. The target population were individuals (≥18-years-old) who recovered from COVID-19 disease. The exclusion criteria included current COVID-19 infections and pre-infection vaccination status. Subjects who had been vaccinated prior to infection were excluded because vaccination strongly influences disease severity and thus may obliterate any effect that could be observed when supplementation was used.

### The study instrument

The study utilized a self-administered questionnaire in the Arabic language. The questionnaire collected information from participants on their demographics (such as age, gender, body mass index [BMI], and education), comorbidities, consumption of supplements (vitamins and minerals), and aspirin use prior to infection. In addition, information about disease symptoms and hospitalization status was collected. The questionnaire was face validated by a group of experts in the field and was piloted on a small sample of the population. Comments were obtained from experts and participants and were used to review and improve the clarity of the questionnaire. Data from the pilot study were not included in the final analyses.

### Sampling procedure

The study followed a convenient sampling procedure. The questionnaire was prepared and administered using Google forms. To ensure anonymity, identifying information such as participants’ names and places of work were not collected. The first part of the questionnaire obtained informed consent and confirmed recovery from COVID-19. To ensure that the Jordanian population is well represented, trained research assistants recruited participants from different Jordanian governorates and assisted the participants in filling out the questionnaire. A total of 2148 participants completed the questionnaire.

### COVID-19 severity classification

Disease symptoms were utilized to classify COVID-19 into two categories as previously reported [22]; severe and non-severe (asymptomatic, mild symptoms, and moderate). Mild cases included fever, sore throat, malaise, body pains, nausea, and other symptoms, but no signs or symptoms of pneumonia. Moderate cases included pneumonia (persistent fever and cough) but no hypoxemia (SpO_2_ ≤ 92%). Severe cases were assigned to those with verified severe pneumonia and hypoxemia [22]. It is worth noting that some severe cases were not admitted to hospitals, which may be due to hospital overcrowding during the peak waves of the COVID-19. Therefore, such cases obtained medical care at home via private doctor visits, and some used medical oxygen supply systems at their homes.

### Statistical analysis

The independent variables in the study were the use of supplements and aspirin. The dependent variables were COVID-19 severity and hospitalization. Frequencies and percentages were used for categorical variables. Means and standard deviations (SD) were used for continuous variables. The Chi-square was used to test differences in the severity/hospitalization between supplement/aspirin usage groups. Multivariate logistic regression analysis was used to explore the effect of supplement/aspirin usage on the severity/hospitalization after adjustment for possible confounders (age, gender, BMI, cigarette smoking, and number of comorbidities). The adjusted odds ratios (OR) and 95% confidence intervals (CI) were reported for each independent variable. A *p* < 0.05 was considered significant. The data were analyzed using the Statistical Package for the Social Sciences version 23 (IBM Inc., Armonk, New York, United States).

## RESULTS

### Characteristics of study participants

Overall, 2148 individuals participated in the study. Table 1 summarizes participants’ characteristics. Females represented 58.2% of the study population. The mean age ± SD of the participants was 40 ± 16 years. Among the participants, 12.1% reported symptoms consistent with severe COVID-19, 10.2% were admitted to the hospital due to COVID-19, 23.1% had at least one comorbidity, and 16.9% were current cigarette smokers (Table 1).

**TABLE 1:**
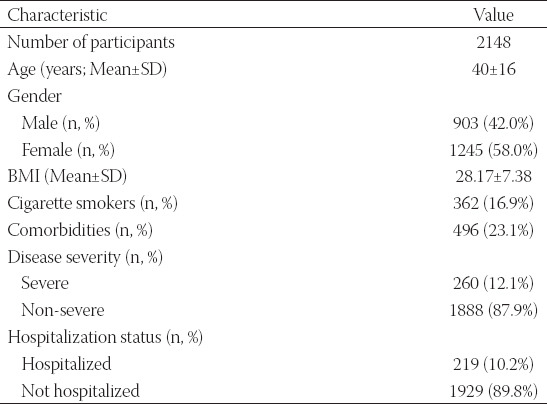
Baseline characteristics of the participants

### Association of supplements and aspirin use with COVID-19 severity and hospitalization

The effects of supplements or aspirin use on the frequencies of COVID-19 severe cases and hospitalizations were investigated (Table 2). Among the supplements included in our study, consumption of vitamin D prior to COVID-19 infection was significantly associated with decreased frequencies of severe illness (*p* = 0.04) and hospitalization (*p* = 0.03). In addition, the rate of hospitalization was significantly decreased among the participants who consumed vitamin C before COVID-19 infection (*p* = 0.03). Vitamin C consumption was also associated with reduced disease severity. However, the difference did not reach statistical significance (*p* = 0.07). Moreover, a significantly higher frequency of severe illness and/or hospitalization was observed among participants who consumed selenium, omega 3, vitamin B complex, calcium, and magnesium supplements, and aspirin before COVID-19 infection (Table 2). On the other hand, there were no significant differences in the frequencies of severe illness and hospitalizations with the consumption of vitamin A, folic acid, vitamin B12, multivitamins, zinc, and iron before COVID-19 infection.

**TABLE 2:**
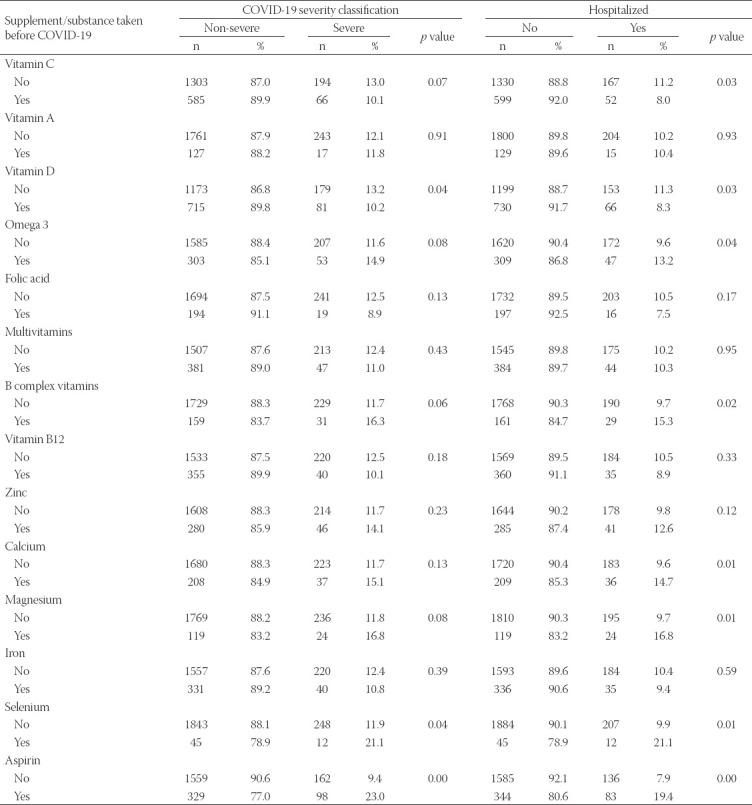
Severe COVID-19 and hospitalization cases according to use of supplements and substances prior to infection

### Logistic regression analyses

Next, to verify the associations above, we performed multivariate logistic regression analyses to control for the possible confounders such as age, gender, BMI, cigarette smoking, and the number of comorbidities. The ORs and 95% CI for the two outcomes, COVID-19 severity, and hospitalization status with the use of each supplement/aspirin, are presented in Table 3. The results of this model demonstrated that after adjusting for the confounders, vitamin D consumption was the only predictive factor to associate with lower risk of COVID-19 severity (OR = 0.68, 95% CI 0.50-0.92; *p* = 0.01, Table 3), and lower percentage of hospitalizations (OR = 0.63, 95% CI 0.45-0.89; *p* = 0.01, Table 3).

**TABLE 3:**
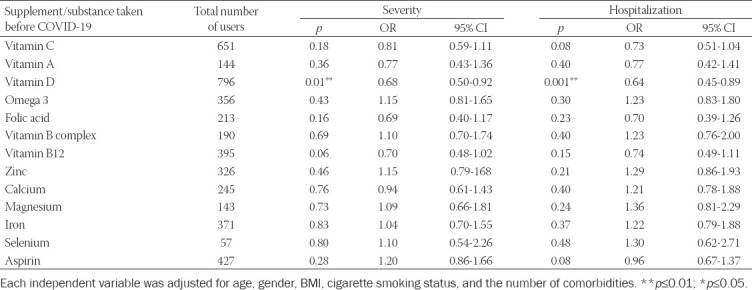
Logistic regression analyses for supplements and aspirin use with COVID-19 severity and hospitalization as the output

## DISCUSSION

In this study, we investigated the impact of using vitamin and mineral supplements and aspirin prior to infection with COVID-19 on disease severity and hospitalization. Previous studies and clinical trials have focused on the impact of using vitamins and minerals during the infection on COVID-19 outcomes [14,23,24]. These studies reported significant effects of several vitamins and trace elements on reducing disease severity [25-27]. However, using these supplements prior to COVID-19 infection can give a powerful boost to the immune system. This increases its effectiveness in the early stages of the disease and could be crucial in determining disease outcome.

In a large survey of 349,598 participants in the UK, vitamin D levels were negatively associated with the probability of COVID-19 infection. After adjusting for confounders in multivariable analyses, this connection was not maintained [28]. In contrast, a large cohort study (n = 108,343) in Spain showed that people with serum vitamin D levels ≥ 30 ng/mL were more likely to have better COVID-19 outcomes after adjusting for outcome variables [29]. In our study, univariate analysis indicated associations between vitamin D and vitamin C consumption and reduced disease COVID-19 severity and hospitalization. However, using regression analysis to adjust for possible confounding factors, only vitamin D consumption was predicted to decrease the risk of severe disease and hospitalization. This indicates that confounding factors likely affected the observed association for vitamin C. Hemilä et al. reported that routine vitamin C supplementation is not justified and fails to reduce the incidence of colds and other respiratory infections [30]. Our findings are consistent with Demir et al., who showed that high vitamin D levels might reduce COVID-19 hospital stay and disease severity [31]. Lower vitamin D levels in COVID-19 patients compared to uninfected individuals were reported previously, suggesting a link between inadequate vitamin D levels and susceptibility to infection [32,33]. In addition, increased infection, hospitalization, and death from COVID-19 have been associated with low vitamin D levels [34-36]. Vitamin D has also been shown to increase the production of anti-inflammatory cytokines, such as IL-10, which is predicted to lower the severity of COVID-19 [37], and improve the activity of macrophages and T lymphocytes [38].

With the exception of vitamin D, adequate amounts of vitamins are obtained through the diet in most individuals [39,40]. However, according to US National Health and Nutrition Examination Survey (NHANES), about half of the respondents (n = 6,261) did not get sufficient vitamin D from food alone, including fortified products [41]. In addition, individuals with darker skin produce less vitamin D than those with lighter skin upon sun exposure [42]. Thus, vitamin D supplementation is necessary for maintaining optimal vitamin D levels. In agreement with our results, a meta-analysis of 25 studies with 11,321 participants in total found that vitamin D supplementation significantly reduced the risk of acute respiratory tract infections [43]. Furthermore, a meta-analysis conducted by Martineau et al. reported that vitamin D supplementation is associated with protection against acute respiratory infections [43]. Therefore, vitamin D supplementation is recommended during the infection with COVID-19, as well as before the infection as a preventative measure [44].

A significantly higher frequency of severe illness and hospitalization was observed among selenium users, omega 3, vitamin B complex, calcium and magnesium supplements, and aspirin. However, none of these supplements were predicted to increase the risk of severity/hospitalization in the logistic regression analyses. This discrepancy can be explained by regular usage of these supplements/aspirin is more frequent among individuals with a higher risk for severe illness such as the elderly and those with pre-existing medical conditions [45,46]. Therefore, controlling for these confounding factors is essential to demonstrate true associations between vitamins, minerals, and aspirin and either disease severity or hospitalization status. Consistent with this conclusion, aspirin intake was not associated with COVID-19-related complications and mortality [47]. Currently, there is no direct evidence to indicate that omega-3 helps in the defense against COVID-19 [48].

Furthermore, logistic regression analysis indicated no association between B vitamins, calcium, and magnesium intake and COVID-19 severity. It is known that B vitamins regulate chemokine and cytokine production and mediate the interplay between immune cells involved in pathological processes [49]. However, the role of B vitamins in reducing COVID-19 severity is highly controversial [50,51]. Similarly, the role of calcium and magnesium body status in COVID-19 manifestations is still controversial [52-54]. It is important to note that the use of supplements such as selenium, vitamin A, and folic acid was too low among the study participants. Therefore, more studies are needed to confirm the present findings.

Although this study assessed the impact of using aspirin and supplements prior to COVID-19 infection on disease severity and hospitalization based on a relatively large sample, it had some limitations. This was a cross-sectional study conducted using an anonymous questionnaire. The findings given were largely self-reported and were dependent on the participants’ reliability, honesty, and recallability. It is likely that some participants answered the questions in a way that they believed was objectively appropriate and not entirely correct. Some participants gave extreme responses based on their personal beliefs introducing so response biases.

Previous literature has shown that the severity of COVID-19 is elevated among the elderly, malnourished individuals, and those with high BMI or comorbidities [55-57]. Therefore, a regression analysis was applied to control for potential confounding factors in the current study. However, it is recommended that the relationship between supplement use and severity be examined in each subgroup with adequate samples in future studies. Among the limitations of the current investigation is that the study did not collect information regarding duration of illness and time of hospitalization. Therefore, the study could not examine the effect of supplement use on such measures. This could be the subject of future investigations. Furthermore, each participant’s rate of supplementary support during the infection was not equal. Furthermore, the levels of supplements and minerals used by the patients were unknown. Finally, since the study is questionnaire-based, fatal cases (that represent the highest severity) could not be included. Thus, more clinical studies are needed to confirm the present findings we are proposing within this study.

## CONCLUSION

Our findings showed that vitamin D supplementation has protective effects against the severity and hospitalization of COVID-19 infection. Therefore, during this pandemic, persons at increased risk of vitamin D insufficiency should consider taking vitamin D supplements to maintain circulating vitamin D at an optimum range.
